# Energy flow analysis of grass carp pond system based on Ecopath model

**DOI:** 10.1007/s11356-023-27154-3

**Published:** 2023-05-09

**Authors:** Shuwen Xiao, Xingguo Liu, Runfeng Zhou, Yuxi Zhao, Zhaoyun Sun

**Affiliations:** 1 Fishery Machinery and Instrument Research Institute, Chinese Academy of Fishery Sciences, Shanghai, 200092 People’s Republic of China; 2 Key Laboratory of Aquaculture Facilities Engineering, Ministry of Agriculture and Rural Affairs, Shanghai, 200092 People’s Republic of China; 3 College of Fisheries and Life Science, Shanghai Ocean University, Shanghai, 201306 People’s Republic of China; 4 Wuxi Fisheries College, Nanjing Agriculture University, Wuxi, 214128 People’s Republic of China

**Keywords:** Ecopath; Grass carp pond, Energy flow, Trophic structure, Monoculture, Polyculture

## Abstract

Grass carp (*Ctenopharyngodon idellus*) is the most productive freshwater fish in China, but its traditional aquaculture model still has problems, such as poor water quality and frequent diseases. We have taken monoculture and 80:20 polyculture grass carp ponds as the research object and used EwE software to build the Ecopath model of two ponds. We analyzed and compared the characteristics of ecological structure and energy flow in two ponds. The result showed the highest effective trophic level in the polyculture pond that was higher than that in the monoculture pond, and fish in polyculture had higher EE values which showed the production of fish in polyculture contributed more to the energy conversion efficiency of the ecosystem. Flows into detritus were the largest component of TST both in the two ponds, which accounted for 49.34% and 50.37%. And the average transfer efficiency in monoculture was 13.07%, while that in polyculture was 15.6%. The ascendency/total development capacity (A/TDC) and overhead/total development capacity (O/TDC) were 0.35 and 0.65 both in the two ponds, respectively, which indicated that both systems had a strong anti-perturbation ability, but the stability could be improved. Finn’s cycling index (FCI) in polyculture was higher and showed that the polyculture pond was more mature and stable. Unused energy of functional groups will flow to detritus, and that in the monoculture pond was higher, the energy of *C. idellus* that flowed to detritus in monoculture was 48.17% higher than that in polyculture; unused energy of bacteria and phytoplankton were also high. The result showed that polyculture could improve energy utilization, increase transfer efficiency, and raise the stability of the ecosystem. Grass carp ponds still need to be improved in the aspects of mixed species and energy consumption. It is necessary to improve the ecological and economic benefits of grass carp ponds by optimizing the aquaculture structure and adjusting the aquaculture proportion.

## Introduction

Grass carp (*Ctenopharyngodon idellus*) is the most productive freshwater fish in China, with an output was 5.75 million tons in 2021 (Bureau of Fisheries Ministry of Agriculture and Rural Affairs et al. 2021). And pond culture accounts for 74% of the total production of grass carp. In the main production areas of China, pond culture of grass carp mainly has two modes: monoculture and polyculture (Chang 2009; Chen and Wang 2016; Yang 2016; Mei and Ren 2017). Monoculture is a culture method with grass carp as a single object. Due to the high breeding density of monoculture, multiple stocking and multiple fishing can obtain higher returns and develop rapidly, but there are some problems, such as complex water quality management and easy disease outbreak (Liu and Jin 1994; Gao et al. 2009; Liu 2012). Polyculture is mainly 80:20 mode (80% of the production is grass carp, and 20% of the production is other fish). The polyculture varieties are mostly silver carp (*Hypophthalmichthys molitrix*), bighead carp (*Aristichthys nobilis*), crucian carp (*Carassius auratus*), and so on (Zhan and Yang 2015). In recent years, with the aggravation of the problem in aquaculture tailwater, this multi-trophic grass carp polyculture model has been widely used. By making full use of the space and resources in the system, the recycling utilization rate of nutrients is improved, to improve the water quality and prevent disease (Dong 2014; Zhou et al. 2016).

However, there are still some problems, such as low feed utilization rate and poor water quality in grass carp ponds, and it is urgent to study the aquaculture structure and material characteristics of the typical grass carp pond systematically. Current research on grass carp ponds focused on carbon, nitrogen, and phosphorus budgets, plankton communities, microbial diversity, productivity, and so on (Song et al. 2011; Zhang et al. 2011; Liu 2012; Tian et al. 2012; Li 2014; Sun et al. 2015). The research on the energy flow and nutrient structure at the ecosystem level in the two common grass carp culture models—monoculture and 80:20 polyculture in China—is still lacking. Comparing and studying the system structure and energy characteristics of different grass carp aquaculture ponds can clarify the flow and transfer transformation characteristics of energy of different trophic levels in aquaculture ponds and find out the methods of reasonable collocation of species in the culture pond system, so as to improve the energy utilization rate in the freshwater pond ecosystem, reduce the waste of energy in the ecosystem, reduce the pollution caused by the discharge of culture wastewater, and improve the efficiency of pond aquaculture.

Based on the principle of nutritional dynamics, the Ecopath model can comprehensively quantify and analyze the structure, material cycle, and energy flow of the ecosystem based on the structure of the ecosystem food webs. And it has been used in aquatic ecosystems such as oceans and lakes. Because pond is a small artificial ecosystem with less biological species, functional groups can be divided into species, which can better understand the complex relationship between species in the system (Zhou et al. 2016). But there are few researches in ponds; only Zhou et al. (2016), Feng et al. (2018), and Zeng et al. (2018) analyzed the grass carp-silver carp-common carp pond, *Portunus trituberculatus-Litopenaeus vannamei-Liza haematocheli* polyculture pond, and compound aquaculture pond, respectively, and established a preliminary evaluation system in ponds.

In this paper, Ecopath with Ecosim software was used to build the Ecopath models of two types of grass carp pond ecosystems: monoculture pond and 80:20 polyculture pond. The specific objectives were as follows: (i) to simulate the nutrient structure and energy flow process of two grass carp ecosystems; (ii) to analyze the ecological characteristics of two aquaculture ponds; (iii) to compare the two ponds and provide ideas for optimizing grass carp ecosystem. It will provide a theoretical basis for evaluating and optimizing the aquaculture system.

## Materials and methods

### Study areas

The experiment was carried out in the Pond Ecological Engineering Research Center of Chinese Academy of Fishery Sciences (Songjiang District, Shanghai, 30.954224 N, 121.158714 E) from May 2021 to March 2022. Two ponds of the same size were selected as the experimental objects, the pond area was 100 m × 50 m, the average water depth was 1.6 m, and the water area was 0.47 hm^2^.

The stocking information of the monoculture pond (G) and 80:20 polyculture pond (GS) shows in Table 1. During the experiment, the management methods of oxygenation and feeding of two ponds were consistent, and there was no fertilization and spraying of insecticides during the aquaculture period.

**Table 1:** Stocking information in different ponds

	G		GS	
	Stocking size (g ind^−1^)	Amount (ind)	Stocking size (g ind^−1^)	Amount (ind)
*C. idellus*	572.24 ± 39.42	1322	495.54 ± 68.76	897
*H. molitrix*	151.62 ± 11.34	277	1252.03 ± 103.21	123
*A. nobilis*	—	—	259.92 ± 31.41	529
*C. auratus*	—	—	94.94 ± 9.43	158
*A. nobilis* fry	—	—	0.5 ± 0.02	2000
*C. auratus* fry	—	—	0.25 ± 0.02	1000

### Model building

#### Modelling approach

A trophic model of grass carp pond was constructed using the Ecopath routine in the Ecopath with Ecosim software. Ecopath divides the ecosystem into functional groups with ecological associations and defines the energy input and output of each functional group as balanced according to the thermodynamic principle (Christensen and Walters 2004):

$$\mathrm{Production}=\mathrm{catches}+\mathrm{predation\ mortality}+\mathrm{biomass\ accumulation}+\mathrm{net\ migration}+\mathrm{other\ mortality}$$  

It can be expressed as,

1 $${P}_{i}={Y}_{i}+{B}_{i}{M2}_{i}+{E}_{i}+{BA}_{i}+{P}_{i}\left(1-{EE}_{i}\right)$$

where *P*_*i*_ is the total production rate of (*i*), *Y*_*i*_ is the total fishery catch rate of (*i*), *M*2_*i*_ is the total predation rate for group (*i*), *B*_*i*_ the biomass of group (*i*), *E*_*i*_ is the net migration rate (emigration–immigration), *BA*_*i*_ is the biomass accumulation rate for (*i*), while *M*0_*i*_ = *P*_*i*_(1 − *EE*_*i*_) is the “other mortality” rate for (*i*).

Equation (1) also can be re-expressed as,

2 $${B}_{i}{\bullet \left(\frac{P}{B}\right)}_{i}-{\sum }_{j}{B}_{j}\bullet {\left(\frac{Q}{B}\right)}_{j}\bullet {DC}_{ji}-{\left(\frac{P}{B}\right)}_{i}\bullet {B}_{i}\left(1-{EE}_{i}\right)-{Y}_{i}-{E}_{i}-{BA}_{i}=0$$

3 $${B}_{i}\bullet {\left(\frac{P}{B}\right)}_{i}\bullet {EE}_{i}-{\sum }_{j}{B}_{j}\bullet {\left(\frac{Q}{B}\right)}_{j}\bullet {DC}_{ji}-{Y}_{i}-{E}_{i}-{BA}_{i}=0$$

where (*P*/*B*)_*i*_ is the production/biomass ratio, (*Q*/*B*)_*j*_ is the consumption/biomass ratio of predator, *DC*_*ji*_ is the fraction of prey (*i*) in the average diet of predator (*j*), and *EE*_*i*_ is the ecotrophic efficiency of (*i*), which can be described as the proportion of the production that is utilized in the system.

#### Functional groups

Functional groups were characterized based on similarities in feeding habits, bulk body mass, life history parameters, physiological behavior, and spatial distributions to keep homogeneous characteristics throughout the species within a group (Milessi et al. 2010). According to the definition and setting principles of functional groups in Ecopath, the monoculture pond and polyculture pond were divided into 16 and 18 ecological functional groups in this study. *C. idellus*, *H. molitrix*, Protozoa, Rotifera, Cladocera, Copepoda, Mollusca, Oligochaeta, Chironomidae, bacteria (water), bacteria (sediment), phytoplankton, feed, precipitation, detritus (water), and detritus (sediment) were in the monoculture pond, and there are more *A. nobilis* and *C. auratu*s in the polyculture pond. Due to the difference in material flow between bacteria and detritus in water and sediment, they were divided into bacteria (water) and bacteria (sediment), and detritus (water) and detritus (sediment). Feed and precipitation only provide energy source for the system, so they were treated as another detritus group. In the model, it is assumed that the unused energy in each functional group flows to detritus (water) and detritus (sediment).

#### Date source

For each functional group, parameters such as biomass (B), production/biomass (P/B), consumption/biomass (Q/B), and diet compositions must be put in the model. Biological samples of each functional groups were taken every 2 months and select three sites for sampling at a time. Biomass (B) and the biomass when calculating P/B and Q/B are the average biomass in the experimental period.

##### Biomass

The biomass (g m^−2^) of all functional groups were measured and calculated through field experiments. Functional groups were sampled every 2 months. Fishes were caught using nets; sampling quantification of plankton and benthos refers to *Water and wastewater monitoring and analysis methods* (State Environmental Protection Administration 1989).

##### Production/biomass

The ratio of *C. idellus*, *H. molitrix*, *A. nobilis*, and *C. auratu*s were calculated from the stocking amount, harvest amount, and monthly average sampling weight; the P/B ratio of Phytoplankton was determined by light and dark bottle technique (Wang 1980); and others were estimated according to the monthly samples and references (Li 2013; Zhou et al. 2016).

##### Consumption/biomass

The Q/B ratios of *C. idellus*, *H. molitrix*, *A. nobilis*, and *C. auratus* were calculated from the stocking amount, harvest amount, feeding amount, and monthly average samples; the ratio of Phytoplankton was determined by light and dark bottle technique; others were estimated according to the monthly samples, P/Q ratio, and references (Liu 1999). The P/Q ratios of zooplankton, Mollusca, Oligochaeta, and Chironomidae are 0.05 (Park 1974; Scavia et al. 1974), 0.125 (Yan and Liang 2003), 0.02, and 0.02 (Halfon et al. 1996). Harvest information is shown in Table 2; feeding amount and average samples are shown in Tables 5 and 6.

**Table 2:** Harvest size in different ponds

	G	GS
	Harvest size (g ind^−1^)	Harvest size (g ind^−1^)
*C. idellus*	1828.85 ± 327.53	1841.25 ± 298.76
*H. molitrix*	945.08 ± 187.91	2279.33 ± 323.21
*A. nobilis*	-	1102.90 ± 439.85
*C. auratus*	-	333.12 ± 107.84

Diet compositions of each functional group were referred to relevant literature (Liu 1999), and Tables 3 and 4 show the diet matrix.

**Table 3:** Diet composition of the monoculture pond

Prey\predator	1	2	3	4	5	6	7	8	9	10	11
*C. idellus*	-	-	-	-	-	-	-	-	-	-	-
*H. molitrix*	-	-	-	-	-	-	-	-	-	-	-
Protozoa	-	0.0060	-	0.0030	0.0190	0.0210	-	-	-	-	-
Rotifera	0.0080	0.0110	-	-	0.0410	0.0950	-	-	-	-	-
Cladocera	0.0080	0.0120	-	-	-	0.1190	-	-	-	-	-
Copepoda	0.0270	0.0190	-	-	-	-	-	-	-	-	-
Mollusca	-	-	-	-	-	-	-	-	-	-	-
Oligochaeta	-	-	-	-	-	-	-	-	-	-	-
Chironomidae	-	-	-	-	-	-	-	-	-	-	-
Bacteria (water)	0.0140	0.0310	0.3010	0.2290	0.1290	0.1200	0.0850	0.1070	0.0970	-	-
Bacteria (sediment)	-	-	0.1410	0.0730	0.0910	0.0390	0.2970	0.3350	0.3010	-	-
Phytoplankton	0.1290	0.6610	0.3370	0.5130	0.5920	0.4570	0.4460	0.4050	0.4360	-	-
Feed	0.7010	0.1010	0.0010	0.0015	0.0017	0.0020	0.0010	0.0010	0.0010	0.0500	0.0500
Precipitation	-	-	0.0010	0.0010	0.0010	0.0010	0.0010	0.0010	0.0010	-	-
Detritus (water)	0.1130	0.1590	0.2110	0.1710	0.1170	0.0910	0.0430	0.0410	0.0510	0.9500	-
Detritus (sediment)	-	-	0.0080	0.0090	0.0080	0.0550	0.1270	0.1100	0.1130	-	0.9500

**Table 4:** Diet composition of the polyculture pond

Prey/predator	1	2	3	4	5	6	7	8	9	10	11	12	13
*C. idellus*	-	-	-	-	-	-	-	-	-	-	-	-	-
*H. molitrix*	-	-	-	-	-	-	-	-	-	-	-	-	-
*A. nobilis*	-	-	-	-	-	-	-	-	-	-	-	-	-
*C. auratus*	-	-	-	-	-	-	-	-	-	-	-	-	-
Protozoa	-	0.0060	0.0050	0.0010	-	0.0030	0.0190	0.0230	-	-	-	-	-
Rotifera	0.0070	0.0110	0.0130	0.0310	-	-	0.0410	0.0970	-	-	-	-	-
Cladocera	0.0080	0.0130	0.0150	0.0370	-	-	-	0.1290	-	-	-	-	-
Copepoda	0.0270	0.0340	0.0350	0.0110	-	-	-	-	-	-	-	-	-
Mollusca	-	-	-	-	-	-	-	-	-	-	-	-	-
Oligochaeta	-	-	-	0.0130	-	-	-	-	-	-	-	-	-
Chironomidae	-	-	-	0.0110	-	-	-	-	-	-	-	-	-
Bacteria (water)	0.0150	0.0370	0.0410	0.0510	0.3130	0.2310	0.1310	0.1200	0.1070	0.1130	0.1030	-	-
Bacteria (sediment)	-	-	-	0.0590	0.1350	0.0710	0.0803	0.0370	0.3150	0.3210	0.3110	-	-
Phytoplankton	0.0870	0.6110	0.6070	0.4130	0.3300	0.5130	0.5910	0.4810	0.4120	0.4530	0.4230	-	-
Feed	0.7550	0.1150	0.1090	0.0710	0.0010	0.0015	0.0017	0.0020	0.0010	0.0010	0.0010	0.0500	0.0500
Precipitation	-	-	-	-	0.0010	0.0010	0.0010	0.0010	0.0010	0.0010	0.0010	-	-
Detritus (water)	0.1010	0.1730	0.1750	0.1310	0.2130	0.1710	0.1280	0.1050	0.0570	0.0340	0.0410	0.9500	-
Detritus (sediment)	-	-	-	0.1710	0.0070	0.0085	0.0070	0.0050	0.1070	0.0770	0.1200	-	0.9500

Unassimilated rate, the internal default value of the model is 0.2, which is for general carnivorous fish according to the references (Park 1974; Halfon et al. 1996; Sun 2020). And the rate of herbivorous fish is 0.41, zooplankton is 0.65, Mollusca is 0.7, and bacteria is 0.4 (Park 1974; Halfon et al. 1996; Sun 2020).

### Model balancing

In the process of model balance debugging, EE (ecotrophic efficiency) < 1 is taken as its basic limiting condition (Christensen et al. 2004). If EE > 1, its diet composition should be adjusted gradually. We check whether the respiration (R) of the functional group is negative; if so, the P/B or Q/B rate should also be adjusted to make the model balanced. Finally, the quality of model was evaluated by Pedigree index; the higher the index, the greater the credibility of the model. If Pedigree index > 0.7, this indicates that the model has high reliability.

## Results

### Basic input and calculated parameters

Tables 5 and 6 show the basic input and calculated parameters for Ecopath model of two ponds. Ecotrophic efficiency (EE) is the fraction of the production used in the system (Christensen and Walters 2004), which is one of the most significant features of an ecosystem and can characterize the conversion efficiency of the contribution of production capacity of each functional group to ecosystem energy. *C. idellus*, *H. molitrix*, Rotifera, Cladocera, and detritus (water) in monoculture pond showed high EE value, while *C. idellus*, *H. molitrix*, *A. nobilis*, *C. auratus*, and Rotifera in the polyculture pond showed high EE value, which were more than 0.9. And the EE of *C. idellus*, *H. molitrix*, Rotifera, Copepoda, Mollusca, Oligochaeta Chironomidae, bacteria (water), and detritus (sediment) in polyculture were higher than monoculture. Phytoplankton both showed low EE value in the two ponds, indicating that the utilization rate of phytoplankton was low.

**Table 5:** Basic input and calculated parameters for Ecopath model of the monoculture pond

Functional groups	Biomass (g m^−2^)	P/B (300 day^−1^)	Q/B (300 day^−1^)	Ecotrophic efficiency	Effective trophic level	Feed import (g m^−2^·300 day^−1^)
*C. idellus*	358.1	1.590	4.210	0.960	2.074	
*H. molitrix*	32.27	1.810	6.710	0.953	2.098	
Protozoa	0.628	82.31	537.6	0.767	2.442	
Rotifera	1.882	77.39	435.9	0.947	2.306	
Cladocera	1.267	108.5	670.5	0.910	2.301	
Copepoda	1.393	198.3	665.7	0.167	2.468	
Mollusca	17.09	1.860	14.88	0.170	2.382	
Oligochaeta	0.124	4.410	220.5	0.226	2.442	
Chironomidae	0.103	8.650	432.5	0.042	2.398	
Bacteria (water)	5.51	114.3	408.5	0.632	2.000	
Bacteria (sediment)	11.75	178.4	545.9	0.158	2.000	
Phytoplankton	24.84	343.8		0.230	1.000	
Feed	1.472			0.941	1.000	1612
Precipitation	0.0104			0.523	1.000	6.24
Detritus (water)	2.615			0.899	1.000	
Detritus (sediment)	2.875			0.504	1.000

**Table 6:** Basic input and calculated parameters for Ecopath model of the polyculture pond

Functional groups	Biomass (g m^−2^)	P/B (300 day^−1^)	Q/B (300 day^−1^)	Ecotrophic efficiency	Effective trophic level	Feed import (g m^−2^·300 day^−1^)
*C. idellus*	240.81	1.640	4.320	0.985	2.075	
*H. molitrix*	46.47	1.310	6.180	0.989	2.127	
*A. nobilis*	95.77	1.700	7.210	0.996	2.137	
*C. auratus*	8.71	1.450	4.880	0.968	2.250	
Protozoa	0.592	62.87	337.4	0.750	2.448	
Rotifera	0.738	127.4	548.0	0.981	2.306	
Cladocera	0.598	203.4	797.0	0.762	2.292	
Copepoda	0.652	250.8	816.7	0.382	2.484	
Mollusca	20.60	4.320	33.84	0.242	2.422	
Oligochaeta	0.1702	5.270	263.5	0.713	2.434	
Chironomidae	0.1362	4.470	223.5	0.790	2.414	
Bacteria (water)	5.19	121.3	327.4	0.679	2.000	
Bacteria (sediment)	13.32	201.5	503.8	0.140	2.000	
Phytoplankton	25.44	341.0		0.213	1.000	
Feed	1.372			0.978	1.000	1351
Precipitation	0.0104			0.382	1.000	6.24
Detritus (water)	2.268			0.716	1.000	
Detritus (sediment)	2.412			0.528	1.000	

### System characteristics

The overall systematic characteristics of the two culture systems are shown in Table 7, which can reflect the scale, stability, and maturity of systems intuitively. TST in the monoculture pond was 34,442.210 g m^−2^·300 day^−1^, higher than 33,058.870 g m^−2^·300 day^−1^ in polyculture. And the TC, TAE, TR, and TD accounted for 39.63%, 1.88%, 9.15%, and 49.34% of TST in monoculture, while they accounted for 38.89%, 2.06%, 8.68%, and 50.37% in polyculture. Flows into detritus were the largest component of TST both in the two ponds. TPP/TR is a crucial indicator of ecosystem maturity, the production will exceed respiration for original accumulation in the early development stage of the system, and it will be greater than 1. TPP/TR in two systems were both more than 1, which showed high primary production capacity. Net system production in monoculture was 5389.220 g m^−2^·300 day^−1^, 7.18% less than that in polyculture. CI and SOI in the monoculture pond were 0.517 and 0.147; they were 0.482 and 0.148, respectively, in the polyculture pond. Two ponds had same value of A/TDC and O/TDC; they were 0.33 and 0.67. FCI of polyculture pond were slightly higher than that of the monoculture pond. The proportion of total flow originating from detritus was high both in the two ponds; they can reach to 67%. EPI of two ponds were 0.724 and 0.765, both more than 0.7 and higher than the numerical range of 0.164 ~ 0.676 in 393 Ecopath models (Morissette 2007), which indicated that models had high reliability.

**Table 7:** Summary statistics of models

Parameter	G	GS	Units
Sum of all consumption (TC)	13,650.120	12,856.550	g m^−2^·300 day^−1^
Sum of all exports (TAE)	648.113	681.517	g m^−2^·300 day^−1^
Sum of all respiratory flows (TR)	3150.027	2868.274	g m^−2^·300 day^−1^
Sum of all flows into detritus (TD)	16,993.950	16,652.530	g m^−2^·300 day^−1^
Total system throughput (TST)	34,442.210	33,058.870	g m^−2^·300 day^−1^
Sum of all production (TP)	12,811.980	13,126.820	g m^−2^·300 day^−1^
Mean trophic level of the catch	2.043	2.086	
Calculated total net primary production (TPP)	8539.246	8674.277	g m^−2^·300 day^−1^
Total primary production/total respiration (TPP/TR)	2.711	3.024	
Net system production	5389.220	5806.003	g m^−2^·300 day^−1^
Total primary production/total biomass (TPP/TB)	18.769	18.890	
Total biomass/total throughput (TB/TST)	0.013	0.014	300 day^−1^
Total biomass (excluding detritus)	454.957	459.197	g m^−2^
Connectance Index (CI)	0.517	0.482	
System omnivory index (SOI)	0.147	0.148	
Ecopath pedigree index (EPI)	0.724	0.765	
Ascendency (A)	52,579	49,320	flowbits m^−2^·300 day^−1^
Overhead (O)	105,171	100,715	flowbits m^−2^·300 day^−1^
Total development capacity (TDC)	157,980	150,176	flowbits m^−2^·300 day^−1^
Ascendency/total development capacity (A/TDC)	0.33	0.33	
Overhead/total development capacity (O/TDC)	0.67	0.67	
Finn’s cycling index (FCI)	29.97	33.63	% of total throughput
Proportion of total flow originating from detritus	69.02	67.75	% of total throughput
Proportion of total flow originating from primary producers	30.98	32.25	% of total throughput

### Ecosystem trophic structure and trophic level

**Fig. 1:**
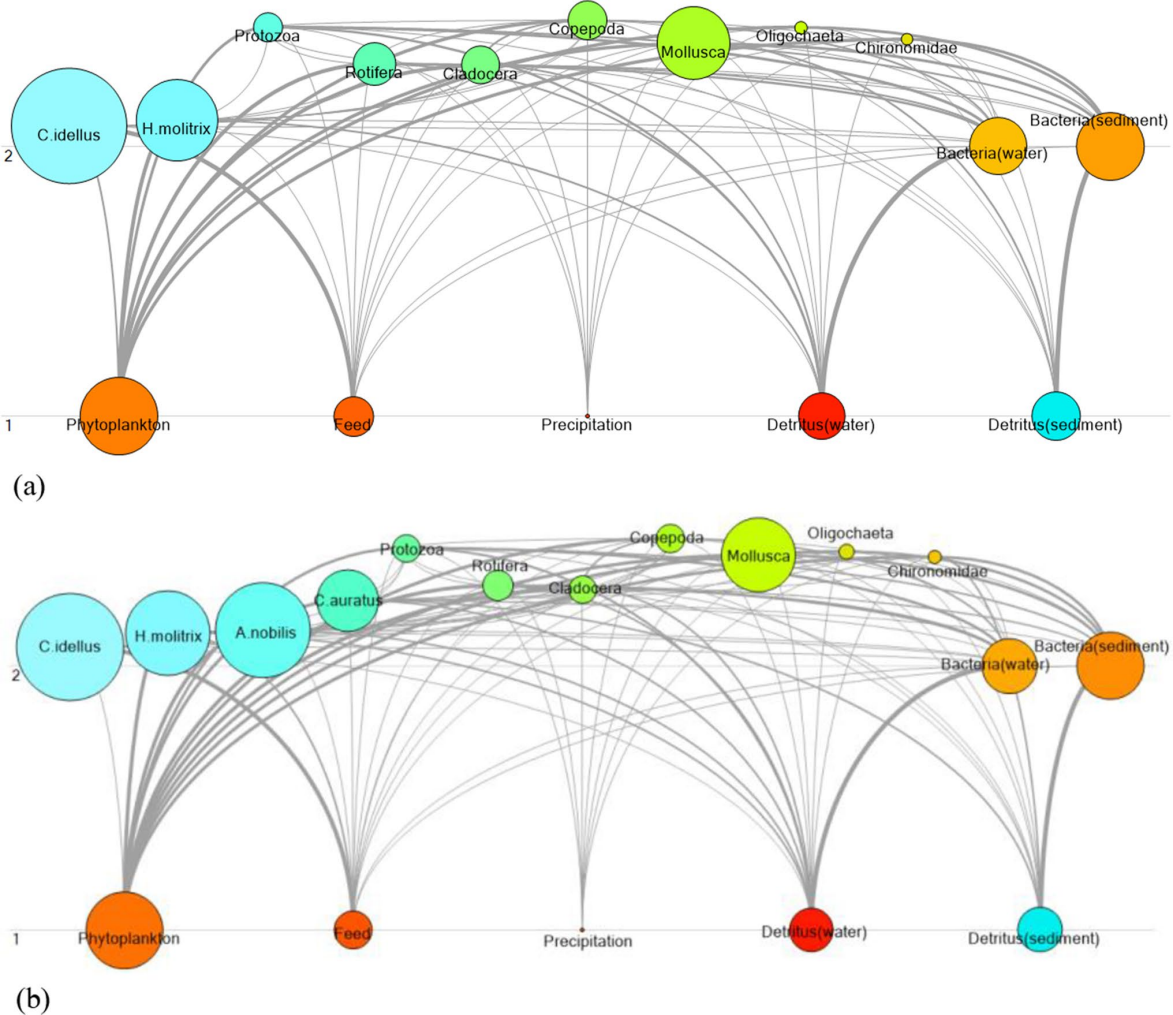
Food wed of the monoculture (**a**) and polyculture (**b**) ponds

Ecopath can calculate the effective trophic level (ETL) of functional groups and present the complex energy flow process of ecosystem. Figure 1 shows the food web of two grass carp ponds. The area of circle represents the relative biomass of functional groups, and the thickness of the line between functional groups represents its proportion in the predator–prey relationship. Tables 5 and 6 show the trophic level of two culture systems. The energy flow of two ponds mainly concentrated on trophic level I and trophic level II, and the phytoplankton, feed, precipitation, and detritus were in trophic level I, which was the start point of energy flow. Bacteria were in trophic level II both in the two ponds, and other functional groups were between trophic level I and II. ETL ranged from 1 to 2.468 in the monoculture pond and ranged from 1 to 2.484 in the polyculture pond. There was little difference in ETL between two ponds; Copepoda was the highest ETL in two systems.

### Energy flow between trophic levels

**Fig. 2:**
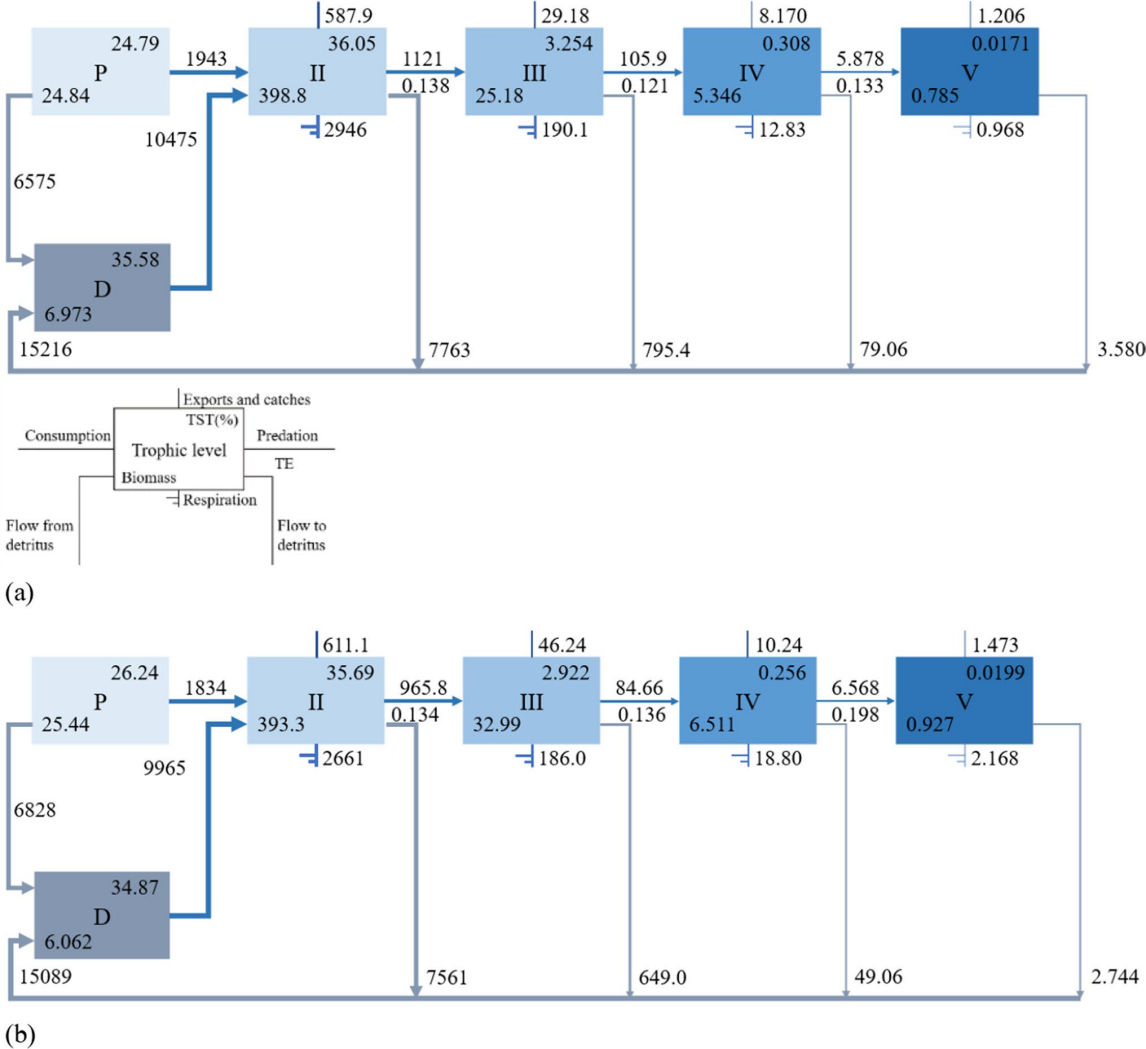
Energy flow among different trophic levels in the monoculture (**a**) and polyculture (**b**) ponds. Note: P is producer, D is detritus, TE is transfer efficiency

Figure 2 shows the energy flow between trophic levels of two models respectively. Trophic level I has the largest throughput in the two ponds, which accounted for 60.37% and 61.11% of TST in monoculture and polyculture. The TST of detritus was larger than producer, and that in monoculture was more than that in polyculture. By comparison, the TST of producer in polyculture was more than that in monoculture. In two ponds, the TST of each trophic level decreased with the increase of trophic level. In monoculture, there was 1943 g m^−2^·300 day^−1^ energy of producer that flowed to trophic level II, and 6575 g m^−2^·300 day^−1^ energy of producer flowed to detritus. And the energy flowed to trophic level II accounted for 22.81% of the throughput in producer. In the polyculture pond, there was 1834 g m^−2^·300 day^−1^ (21.17%) energy of producer that flowed to trophic level II, and 6828 g m^−2^·300 day^−1^ energy of producer flowed to detritus. TE of each trophic level is between 10 and 20% (Lindeman 1991).

### Consumptions of energy by consumer

During the aquaculture period, the proportion of energy consumed by consumers is shown in Table 8. Bacteria (sediment) was the functional group with the largest energy consumption both in the two ponds, which account for 46.99% and 52.20%. In the monoculture pond, the energy consumption ratios of *C. idellus* and *H. molitrix* were 11.04% and 1.59%, which accounted for 12.63%. In polyculture pond, the energy consumption ratios of *C. idellus*, *H. molitrix*, *A. nobilis*, and *C. auratus* were 8.29%, 2.23%, 5.37%, and 0.33%, which account for 16.03% in all. And the energy consumption ratio of zooplankton accounted for 21.50% and 12.55% respectively in the two ponds, and the benthos accounted for 1.86% and 6.01%. The energy consumption of fish was low.

**Table 8:** Proportion of energy throughput consumption in the monoculture (a) and polyculture (b) pond

Group name	G	GS
*C. idellus*	11.04%	8.09%
*H. molitrix*	1.59%	2.23%
*A. nobilis*	-	5.37%
*C. auratus*	-	0.33%
Protozoa	2.47%	1.55%
Rotifera	6.01%	3.15%
Cladocera	6.22%	3.71%
Copepoda	6.79%	4.14%
Mollusca	1.86%	5.42%
Oligochaeta	0.20%	0.35%
Chironomidae	0.33%	0.24%
Bacteria (water)	16.49%	13.22%
Bacteria (sediment)	46.99%	52.20%

### Energy flow of detritus

The energy flowed to detritus and the proportion of flow from detritus in the two ponds are shown in Tables 9 and 10. The proportion of *C. idellus* flowed from detritus was 0.8460 and 0.8880, which indicated that feed played an important role in its diet composition. The energy flowed to detritus was 15,375.7125 g m^−2^·300 day^−1^ in monoculture, which was slightly higher than that in the polyculture pond (15,295.2863 g m^−2^·300 day^−1^) and showed that unused energy in monoculture was higher. Among them, the energy in *C. idellus* flowed to detritus was 640.6955 g m^−2^·300 day^−1^ in monoculture, which was 48.17% higher than that in polyculture.

**Table 9:** The energy flowed to detritus and the proportion of energy flowed from detritus in monoculture

Group name	Flow to detritus (g m^−2^·300 day^−1^)	Proportion of flow from detritus
*C. idellus*	640.6955	0.8460
*H. molitrix*	46.0251	0.3130
Protozoa	231.4934	0.6630
Rotifera	540.9368	0.4860
Cladocera	564.6221	0.3800
Copepoda	832.4828	0.4130
Mollusca	204.3858	0.5540
Oligochaeta	5.9058	0.5950
Chironomidae	9.7635	0.5640
Bacteria(water)	1233.5520	1.0000
Bacteria(sediment)	4331.1210	1.0000
Phytoplankton	6574.8880	0.0000
Feed	94.8510	0.0000
Precipitation	2.9792	0.0000
Detritus(water)	62.0107	0.0000

**Table 10:** The energy flowed to detritus and the proportion of energy flowed from detritus in polyculture

Group name	Flow to detritus (g m^−2^·300 day^−1^)	Proportion of flow from detritus
*C. idellus*	432.4011	0.8880
*H. molitrix*	58.1326	0.3520
*A. nobilis*	138.8294	0.3540
*C. auratus*	8.9005	0.5310
Protozoa	139.1608	0.6700
Rotifera	264.6361	0.4860
Cladocera	338.6520	0.3820
Copepoda	436.8808	0.3820
Mollusca	555.4048	0.5880
Oligochaeta	9.2271	0.5470
Chironomidae	6.2158	0.5770
Bacteria (water)	881.6356	1.0000
Bacteria (sediment)	4991.8400	1.0000
Phytoplankton	6827.6130	0.0000
Feed	30.2926	0.0000
Precipitation	3.8543	0.0000
Detritus (water)	171.6098	0.0000

### Niche overlap

**Fig. 3:**
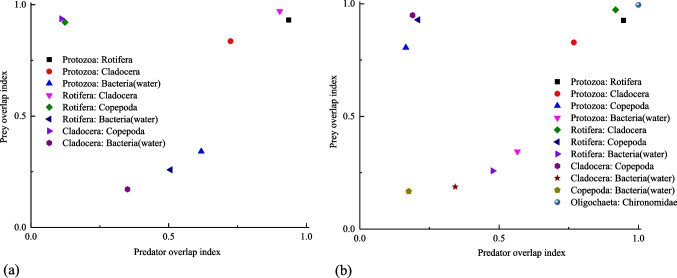
Niche overlap plot in the monoculture (**a**) and polyculture (**b**) ponds

Prey overlap index and predator overlap index can used to analyze niche overlap in Ecopath (Christensen et al. 2004). Prey overlap index can reflect the similarity of diet composition between different functional groups, which was used to analyze intensity of prey competition by predators. And predator overlap index can used to analyze whether there were similar predators between functional groups. The closer the index is to 1, the greater niche overlap. In the monoculture pond, the prey overlap indexes of Protozoa:Rotifera, Rotifera:Cladocera, Rotifera:Copepoda, and Cladocera:Copepoda were more than 0.9 (Fig. 3a), which shown that they had similar diet composition. Predator overlap indexes of Protozoa:Rotifera, Protozoa:Cladocera, and Rotifera:Cladocera were more than 0.7, indicated they were caught by similar predators. And Protozoa:Rotifera, Rotifera:Cladocera had similar diet composition and predators. In the polyculture pond (Fig. 3b), the prey overlap index and predator overlap index of Protozoa:Rotifera, Protozoa:Cladocera, Rotifera:Cladocera, and Oligochaeta:Chironomidae were all more than 0.7, which indicated that Protozoa and Rotifera, Protozoa and Cladocera, Rotifera and Cladocera, and Oligochaeta and Chironomidae had similar prey and were ingested by similar predators.

### Analysis of mixed trophic impact

**Fig. 4:**
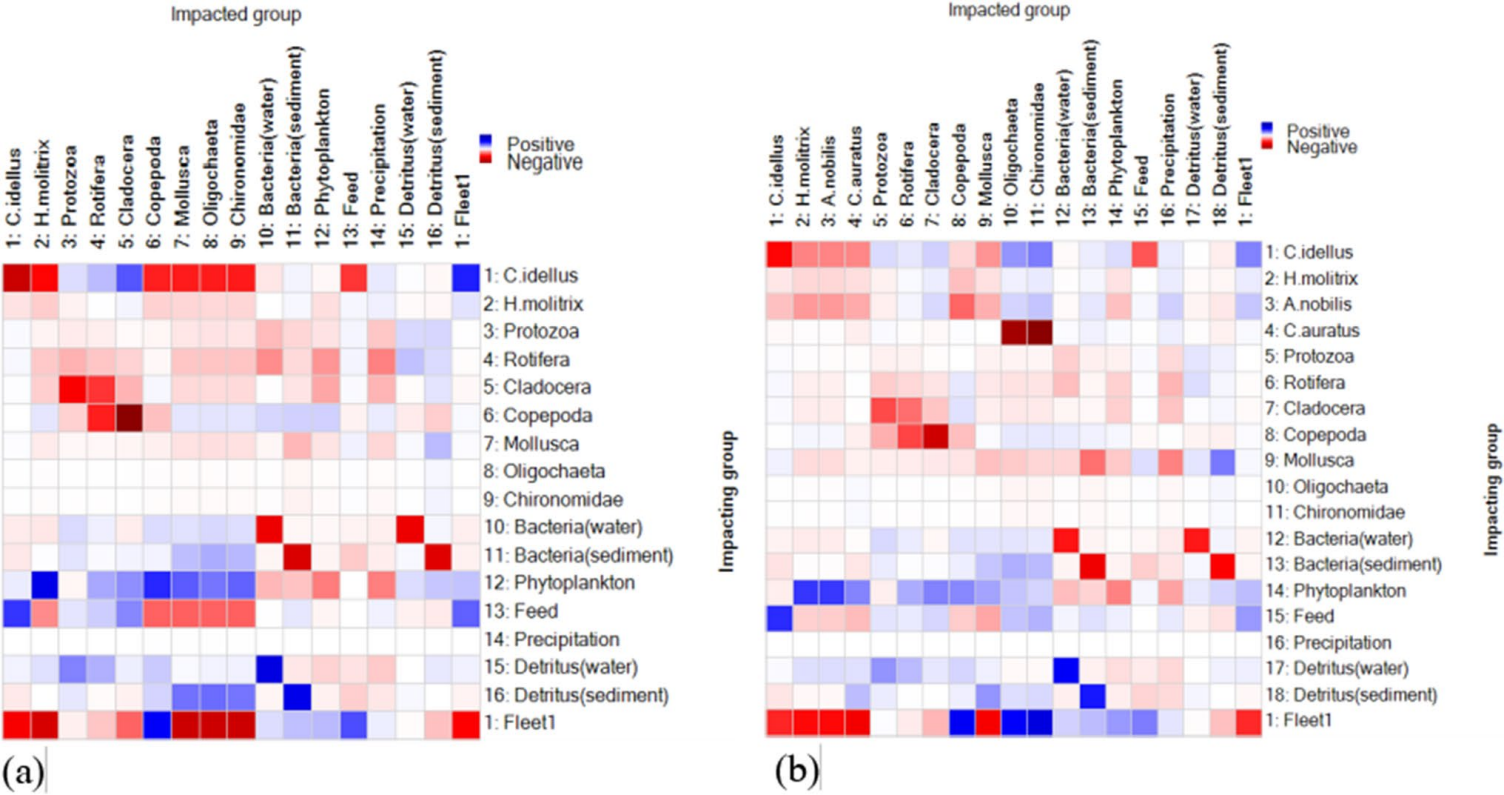
Mix trophic impact in monoculture (**a**) and polyculture (**b**) ponds

Mix trophic impact (MTI) is shown in Fig. 4. The depth of color is proportional to the influence between function groups. Most functional groups showed negative effects on themselves except Oligochaeta, Chironomidae, bacteria, feed, precipitation, and detritus, which reflected the density restriction effect within each functional group. In monoculture, Copepoda showed a strong negative impact on Cladocera, and the main reason should be they had similar diet composition and predation impact. The functional groups in trophic level I showed a positive impact on most of other functional groups in both two ponds. In polyculture pond, Copepoda also showed a strong negative impact on Cladocera. And *C. auratus* had a strong negative impact on Oligochaeta and Chironomidae because of the predation impact.

## Discussion

### Evaluation of parameters in culture systems

Input data of two models obtained through sampling and calculating P/Q and diet composition is referred to in the literature. These values are hard to determine in the field (Calderon-Aguilera et al. 2021) and would not have significantly altered the system’s biomass budget in most cases (Lin et al. 2006). Ecopath pedigree indexes in the two ponds were 0.724 and 0.765, which showed that models had high reliability, which can reflect the actual situation of grass carp ponds. EE in the monoculture pond is between 0.042 and 0.960, and that in the polyculture ponds is between 0.140 to 0.996. Normally, functional groups just over the zero-value showed they were not consumed until now, while close to 1 designates that heavily preyed and caught (Karim et al. 2019). Fish in two ponds both had high EE, which was similar to that of the grass carp-silver carp-common carp polycultured pond studied by Zhou et al. (2016), and higher than that of the grass carp-silver carp-white shrimp polycultured system studied by Bao et al. (2013). As the main product in the process of aquaculture, fish were fully caught at the end of aquaculture, so they had high EE values. The EE values of fish in polyculture were higher than that in monoculture, which indicated that the conversion efficiency of the energy contribution of fish in the polyculture pond to the ecosystem is high. The functio”al g’oups with high predation pressure also had high EE values, like zooplankton and feed. Different from other grass carp ponds, the EE values of phytoplankton in the experimental ponds were lower, which may be caused by the high biomass of phytoplankton in the two ponds and the lack of corresponding predators. The EE value of detritus (water) in monoculture was higher than that in polyculture, while the EE value of detritus (sediment) was less than that in polyculture, and the EE value of detritus in both two ponds was higher than that of some natural aquatic systems such as Qiandao Lake (Wang et al. 2019; Deng et al. 2022). Most of the detritus in the two ponds recycled and reused into the food web. The recycling utilization rate of detritus (water) in monoculture was higher, and the recycling utilization rate of detritus (sediment) in polyculture was higher, which is related to the addition of crucian carp and more benthos in the polyculture pond.

TPP/TR in polyculture was 3.024 and higher than that in the monoculture pond (2.711), which was higher than grass carp-silver carp-common carp polyculture system (1.43) (Zhou 2015), but lower than Qiandao Lake (Deng et al. 2022), which showed that the polyculture pond has higher development potential (Ludovisi et al. 2005; Wang et al. 2019; Deng et al. 2022). Connection index (CI) and system omnivory index (SOI) can reflect the complexity of the food web in the ecosystem to some extent. In this experiment, the CI and SOI of the two grass carp ponds were higher than those of other aquaculture ponds (Feng et al. 2018; Hu 2020), and also higher than the general natural ecosystem (Jia 2012; Wang et al. 2019; Deng et al. 2022); this may be influenced by manual intervention (Zeng et al. 2018). Ascendency/total development capacity (A/TDC) represents the stability of the energy flow within an ecosystem, while overhead/total development capacity (O/TDC) shows the ability of a system to maintain system stability when subjected to external interference. Compared with other aquaculture ponds, the two grass carp ponds had higher O/TDC, which showed strong anti-interference ability. The two systems also had high Finn’s cycling index (FCI), which was higher than other grass carp ponds studied by Zhou (2015) and *Portunus trituberculatus-Litopenaeus vannamei-Liza haematocheli* polyculture ponds studied by Feng et al. (2018). FCI strongly correlates with system maturity, resilience, and stability (Odum 1969; Christensen et al. 2004); it also represents the ratio between the energy flow of the detritus into the culture system and the total energy flow of the system (Finn 1976). The higher the FCI, the higher the nutrient recycling rate (Zhao 2022), and the stronger the anti-interference of the system (Christensen and Pauly 1993; Vasconcellos et al. 1997). And the polyculture will increase the paths of energy flow, improve the recycling rate of nutrients, and increase the stability and anti-interference ability of the aquaculture system.

### Characteristics of energy flow in aquaculture systems

Both ponds contained two nutrient circulation paths: grazing food chain starting from phytoplankton and detrital food chain starting from detritus. In terms of the nutrient flow rate, the throughput of detrital food chain was greater than grazing food chain both in two ecosystems, 69.02% of energy in the monoculture pond was input from the detrital food chain, while 67.75% energy in polyculture was input. Similar to other aquaculture ponds (Zhou 2015; Feng et al. 2018; Hu 2020; Zhao 2022), detrital food chain played a greater role on cultured organisms in the two grass carp ponds; it may be caused by the input of feed increases the feeding pressure of the system on the detritus functional group (Lassalle et al. 2013). The average of transfer efficiency (TE) in monoculture was 13.07%, while that in polyculture was 15.6%. They were similar to other aquaculture ponds and both between the average energy transfer efficiency of the natural system (Lindeman 1991; Feng et al. 2018), and the TE in polyculture was higher than monoculture. The highest effective trophic level in monoculture was 2.468, and that in polyculture was slightly higher, which was 2.484. They were both lower than other natural water ecosystems, because the feed was the main diet composition and there were few species in grass carp ponds (Lin et al. 2007; Liu et al. 2007; Zhou et al. 2016). Predators with high trophic level can couple energy from different energy sources of the system by ingesting, which is an important factor in maintaining the stability of an ecosystem (Rooney et al. 2006). Because the ETL were not high, the SOI of systems were lower. Unused energy of functional groups will flow to detritus, and the unused energy in bacteria and phytoplankton were more, indicating that much of the energy in them is not ingested by the relevant predators (Dong et al. 2021). This is similar to the *Portunus trituberculatus-Litopenaeus vannamei-Liza haematocheli* polyculture ponds (Feng et al. 2018); most energy in phytoplankton and bacteria flows into detritus due to the lack of predators. As filter feeding fish such as silver carp and bighead carp are added to the polyculture pond, the feeding pressure on zooplankton will increased, so the energy of zooplankton flowing to detritus will reduce, while zooplankton in monoculture had more unused energy (Feng et al. 2018). And polyculture can increase food chains by enriching species, increase the energy utilization, get higher TE, and reduce the unused energy flowing to detritus.

### Optimization of aquaculture structure

Similar to other grass carp ponds, the main sources of detritus in the aquaculture ecosystem are zooplankton and bacteria (Zhou 2015). Reducing the proportion of detritus flowing into the pond can increase the energy utilization rate of the system. In the monoculture pond, *C. idellus* had negative impact on *H. molitrix*, because they had slightly predation overlaps. Reducing the number of *C. idellus* appropriately and increasing the input of *H. molitrix* can reduce the adverse effects. And stocking *C. auratus* can increase the ingestion and utilization of detritus (sediment) and benthos; it also can reduce the negative impact of feed on benthos. Energy of phytoplankton flowed to detritus accounted for 42.76% and 44.64%; stocking some shrimp and barracuda to increase the utilization of phytoplankton is necessary (Feng et al. 2018). Energy of zooplankton flowed to detritus accounted for 14.11% and 7.71%, which showed that the input of *A. nobilis* and *C. auratus* can reduce the waste of energy. Putting some fish feeding on Mollusca also can improve the energy utilization of the system.

## Conclusion

This study analyzed and compared the characteristics of energy flow in monoculture and 80:20 polyculture grass carp ponds. The result showed that polyculture could improve the EE value of fish, reduce the waste of energy, increase the transfer efficiency, and raise the stability of the ecosystem. However, it also has some problems such as the insufficient utilization of phytoplankton and bacteria and the trophic structure is simple because of the small culture scale. It is necessary to optimize the aquaculture structure and adjust the aquaculture proportion by stocking omnivorous fish and shrimp, to exert the production potential of the system and improve the economic and ecological benefits of grass carp aquaculture system.

## Data Availability

The datasets used or analyzed during the current study are available by emailing 949,960,731@qq.com on reasonable request.

## References

[CR1] Bao W-Y, Yang M-Y, Liu X-T, Shan H-W, Wang F (2013) Ecotrophic efficiency comparison of three culture modes of grass carp based on analyses of Ecopath with Ecosism. International Conference on Materials, Architecture and Engineering Technology, pp 300–306

[CR2] Bureau of Fisheries Ministry of Agriculture and Rural Affairs, National Fisheries Technology Extension Center, China Society of Fisheries (2021). 2021 China fishery statistics yearbook.

[CR3] Calderon-Aguilera LE, Reyes-Bonilla H, Olan-Gonzalez M, Castaneda-Rivero FR, Perusquia-Ardon JC (2021) Estimated flows and biomass in a no-take coral reef from the eastern tropical Pacific through network analysis. Ecol Indic 123. 10.1016/j.ecolind.2021.107359

[CR4] Chang H (2009) General situation and present situation analysis of grass carp culture in Guangdong province. Ocean and Fishery 6:50–52

[CR5] Chen X-J, Wang Q-C (2016). Problems and countermeasures of grass carp culture pattern in Tonghaikou area of Hubei Province. Fishery Guide to Be Rich.

[CR6] Christensen V, Pauly D (1993) Trophic models of aquatic ecosystems. International Center for Living Aquatic Resources Management, International Council for the Exploration of the Sea, Danish International Development Agency

[CR7] Christensen V, Walters C, Pauly D (2004). Ecopath with Ecosim: a user’s guide. Fisheries Centre Research Reports.

[CR8] Christensen V, Walters CJ (2004). Ecopath with Ecosim: methods, capabilities and limitations. Ecol Model.

[CR9] Deng Y, Zheng Y-C, Chang J-B (2022). Evaluation of the effect of stocking silver carp and bighead carp on the ecosystem of Qindao Lake using Ecopath model. Acta Ecol Sin.

[CR10] Dong S-L (2014). Ecological basis of integrated aquaculture in China.

[CR11] Dong S-P, Gao Y-F, Gao Y-P, He M-D, Liu F, Yan F-J, Wang F (2021) Evaluation of the trophic structure and energy flow of a rice-crayfish integrated farming ecosystem based on the Ecopath model. Aquaculture 539. 10.1016/j.aquaculture.2021.736626

[CR12] Feng J, Tian X-L, Dong S-L (2018). Energy flux analysis of *Portunus**trituberculatus-Litopenaeus**vannamei**-Liza **haematocheli* polyculture system based on EwE model. Periodic Ocean Univ China.

[CR13] Finn JT (1976). Measures of ecosystem structure and function derived from analysis of flows. J Theor Biol.

[CR14] Gao P, Jiang M, Zhao Y-J, Wu F, Liu W, Leng X-J, Wen H (2009). Variation rules of water quality and budget of nitrogen and phosphorus in ponds with grass carp as the dominant cultured species. J Yunnan Agric Univ.

[CR15] Halfon E, Schito N, Ulanowicz RE (1996). Energy flow through the Lake Ontario food web: conceptual model and an attempt at mass balance. Ecol Model.

[CR16] Hu G-Y (2020). Research on integrated aquaculture mode of shrimp and shellfish pond based on Ecopath model.

[CR17] Jia P-Q (2012). Ecopath model construction and ecological effect of pen culture of silver carp and bighead carp in Gehu Lake College of Fisheries and Life Science.

[CR18] Karim E, Liu Q, Xue Y, Hasan SJ, Hoq ME, Mahmud Y (2019). Trophic structure and energy flow of the resettled maritime area of the Bay of Bengal, Bangladesh through ECOPATH. Acta Oceanol Sin.

[CR19] Lassalle G, Lobry J, Le Loc’h F, Mackinson S, Sanchez F, Tomczak MT, Niquil N (2013). Ecosystem status and functioning: searching for rules of thumb using an intersite comparison of food-web models of Northeast Atlantic continental shelves. Ices J Mar Sci.

[CR20] Li B (2013). A preliminary study on bacterial productivity in different grass carp polyculture eco-systems and analysis of bacterial community.

[CR21] Li R-J (2014). Studies on phytoplankton community structure and its primary productivity, energy conversion efficiency in the ponds stocked mainly of *Ctenopharyngodon**idellus*.

[CR22] Lin H-J, Dai X-X, Shao K-T, Su H-M, Lo W-T, Hsieh H-L, Fang L-S, Hung J-J (2006). Trophic structure and functioning in a eutrophic and poorly flushed lagoon in southwestern Taiwan. Mar Environ Res.

[CR23] Lin H-J, Shao K-T, Jan R-Q, Hsieh H-L, Chen C-P, Hsieh L-Y, Hsiao Y-T (2007). A trophic model for the Danshuei River Estuary, a hypoxic estuary in northern Taiwan. Mar Pollut Bull.

[CR24] Lindeman RL (1991). The trophic-dynamic aspect of ecology. Bull Math Biol.

[CR25] Liu J-K (1999). Advanced hydrobiology.

[CR26] Liu L-Y, Jin Y-K (1994). Preliminary investigation on hydrochemical environment of fish pond water during outbreak of bacterial septicemia in cultured fishes. J Fisheries China.

[CR27] Liu P (2012). A preliminary study on budget and variation of organic carbon, nitrogen and phosphorous of grass carp in different polyculture systems.

[CR28] Liu Q-G, Chen Y, Li J-L, Chen L-Q (2007). The food web structure and ecosystem properties of a filter-feeding carps dominated deep reservoir ecosystem. Ecol Model.

[CR29] Ludovisi A, Pandolfi P, Taticchi MI (2005). The strategy of ecosystem development: specific dissipation as an indicator of ecosystem maturity. J Theor Biol.

[CR30] Mei S, Ren Q-S (2017). Investigation and study on practical breeding model of grass carp in Nanling County. Modern Agric Technol.

[CR31] Milessi AC, Danilo C, Laura RG, Daniel C, Sellanes J, Rodriguez-Gallego L (2010). Trophic mass-balance model of a subtropical coastal lagoon, including a comparison with a stable isotope analysis of the food-web. Ecol Model.

[CR32] Morissette L (2007) Complexity, cost and quality of ecosystem models and their impact on resilience: a comparative analysis, with emphasis on marine mammals and the Gulf of St. Lawrence. University of British Columbia, Vancouver

[CR33] Odum EP (1969). The strategy of ecosystem development. Science.

[CR34] Park RA (1974). A generalized model for simulating lake ecosystems. Simulation.

[CR35] Rooney N, McCann K, Gellner G, Moore JC (2006). Structural asymmetry and the stability of diverse food webs. Nature.

[CR36] Scavia D, Bloomfield JA, Fisher JS, Nagy J, Park RA (1974). Documentation of CLEANX: a generalized model for simulating the open-water ecosystems of lakes. Simulation.

[CR37] Song Q, Tian X-L, Dong S-L, Wang F, Zhang Z-D (2011) An experiment study on the energy budget and conversion efficiency of ecosystem in polyculture of *Ctenopharyngodon idellus*. Periodic Ocean Univ China 41:45–51

[CR38] State Environmental Protection Administration (1989). Water and wastewater monitoring and analysis methods.

[CR39] Sun N-N (2020). Evaluation of water environment quality and construction of Ecopath model in Xidayang reservoir ecological restoration project demonstration area College of Fisheries and Life Science.

[CR40] Sun Y-F, Wang F, Liu F, Dong S-L (2015). Nitrogen and phosphorus budget of grass carp, silver carp and common carp in different polyculture systems. J Fish Sci China.

[CR41] Tian X-L, Zheng Y-Y, Liu B-J, Wang F, Dong S-L (2012). Abundance dynamics and community functional diversity of bacteria in grass carp polyculture systems. Periodic Ocean Univ China.

[CR42] Vasconcellos M, Mackinson S, Sloman K, Pauly D (1997). The stability of trophic mass-balance models of marine ecosystems: a comparative analysis. Ecol Model.

[CR43] Wang J (1980). Primary productivity of phytoplankton and oxygen measurement in black and white bottle. Freshwater Fisheries.

[CR44] Wang W, Wang J-P, Zuo P, Li Y, Zou X-Q (2019). Analysis of structure and energy flow in southwestern Yellow Sea ecosystem based on Ecopath model. J Appl Oceanogr.

[CR45] Yan Y-J, Liang Y-L (2003) Energy flow of macrozoobenthic community in a macrophytic lake, Biandantang Lake. Acta Ecol Sin 23(3):527–538

[CR46] Yang F (2016). Hubei grass carp culture model summary of The New Continent. Curr Fish.

[CR47] Zeng X-L, Wei B-C, Liu X-G, Gu Z-J, Lu S-M, Che X (2018). Analysis of compound culturing pond build based on Ecopath model. J Fish China.

[CR48] Zhan J-Z, Yang Q (2015). Efficient freshwater fish farming.

[CR49] Zhang Z-D, Wang F, Dong S-L, Gao Q-F, Zhang M-Z, Song Q, Zhang J-D (2011). A preliminary study on structural optimization in polycultural systems of *Ctenopharyngodon**idellus* with *Hypophthalmichthys** molitrix* and *Litopenaeus**vannamei*. Periodic Ocean Univ China.

[CR50] Zhao Y-X (2022). Investigation on integrated multi-trophic light-controlled aquaculture system of *Pelteobagrus**fulvidraco* College of Fisheries and Life Science.

[CR51] Zhou B (2015). Research on grass carp (*Ctenopharyngodon**idella*) integrated aquaculture pond ecosystem: based on EwE model.

[CR52] Zhou B, Dong S-L, Wang F (2016) Modeling analysis of the structure of grass carp-silver carp-common carp polycultured pond ecosystem. Periodic Ocean Univ China 46(4):28–36

